# Procedural Feasibility and Long-Term Efficacy of Catheter Ablation of Atypical Atrial Flutters in a Wide Spectrum of Heart Diseases: An Updated Clinical Overview

**DOI:** 10.3390/jcm11123323

**Published:** 2022-06-09

**Authors:** Roberto De Ponti, Raffaella Marazzi, Manola Vilotta, Fabio Angeli, Jacopo Marazzato

**Affiliations:** 1 Department of Medicine and Surgery, University of Insubria, 21100 Varese, Italy; j.marazzato88@gmail.com; 2 Department of Heart and Vessels, Ospedale di Circolo, 21100 Varese, Italy; raffaella.marazzi@gmail.com (R.M.); manolavilotta@gmail.com (M.V.); 3 Department of Medicine and Cardiopulmonary Rehabilitation, Maugeri Care and Research Institute, IRCCS Tradate, 21049 Tradate, Italy; angeli.internet@gmail.com

**Keywords:** atypical atrial flutter, atrial fibrillation, catheter ablation

## Abstract

Atypical atrial flutters (AAFL) are difficult-to-manage atrial arrhythmias, yet potentially amenable to effective radiofrequency catheter ablation (CA). However, data on CA feasibility are only sparingly reported in the literature in different clinical settings, such as AAFL related to surgical correction of congenital heart disease. The aim of this review was to provide an overview of the clinical settings in which AAFL may occur to help the cardiac electrophysiologist in the prediction of the tachycardia circuit location before CA. Moreover, the role and proper implementation of cutting-edge technologies in this setting were investigated as well as which procedural and clinical factors are associated with long-term failure to maintain sinus rhythm (SR) to find out which patients may, or may not, benefit from this procedure. Not only different surgical and non-surgical scenarios are associated with peculiar anatomical location of AAFL, but we also found that CA of AAFL is generally feasible. The success rate may be as low as 50% in surgically corrected congenital heart disease (CHD) patients but up to about 90% on average after pulmonary vein isolation (PVI) or in patients without structural heart disease. Over the years, the progressive implementation of three-dimensional mapping systems and high-density mapping tools has also proved helpful for ablation of these macro-reentrant circuits. However, the long-term maintenance of SR may still be suboptimal due to the progressive electroanatomic atrial remodeling occurring after cardiac surgery or other interventional procedures, thus limiting the likelihood of successful ablation in specific clinical settings.

## 1. Introduction

Atypical atrial flutters (AAFL) are complex cardiac arrhythmias that often involve re-entrant circuits related to atrial scarring and areas of slow conduction [1]. They may arise from the right as well the left atrial chamber [1]. Differently from the typical atrial flutter (AFL) where the electrical wavefront revolves around the cavotricuspid isthmus, the key to diagnosing AAFL is the reconstruction of the re-entry course by mapping and clear identification of the area critical to the re-entrant circuit.

AAFL may be observed in different clinical scenarios [1] spanning from patients who have undergone surgical correction of congenital [2] and acquired valvular heart disorders [3] to patients that have underwent non-surgical ablation of atrial fibrillation (AF) [4] and may even occur in apparently normal hearts [5].

Whatever the underlying structural heart disease, the greater the complexity of the pathophysiological substrate, such as in case of intra-atrial reentrant tachycardias (IART) in patients with congenital heart disease (CHD), the more difficult the clinical management. In fact, by promoting slow conduction through ion channel blockade, antiarrhythmic drugs (AAD) may even paradoxically enhance the risk of arrhythmia maintenance with potentially troublesome clinical consequences in the affected patients [6]. For this reason, catheter ablation (CA) has emerged as a potentially definitive treatment option for palpitations, heart failure, and even sudden cardiac death [2]. However, the associated long-term maintenance of sinus rhythm (SR) may be disappointing despite the implementation of cutting-edge technologies in this field [7].

Thus, the aim of this review was to explore the wide spectrum of clinical settings in which AAFL may occur and to provide the cardiac electrophysiologist with a better understanding of common arrhythmia locations and associated arrhythmogenic substrates in all these scenarios. Further, we evaluated the methodologies (e.g., the utility of cutting-edge technologies in the field) and the acute and long-term outcomes associated with CA of AAFL. The factors connected with both periprocedural and long-term failure to maintain SR were also investigated. With this review we hope to elucidate ways to improve the overall procedural efficacy in this complex clinical scenario.

## 2. Clinical Settings Associated with Atypical Atrial Flutters

### 2.1. Surgical Correction for Congenital Heart Disease

Macro-rentrant atrial arrhythmias or post-incisional IART represent common complications after surgical correction for congenital heart disease [8]. IART generally develops in adulthood several years after surgery and is often poorly tolerated in these patients [2]. Cavo-tricuspid isthmus-dependent AFL is seen in at least 58% of patients after cardiac surgery [2,9], whereas IART occurs in up to 25% of cases [2]. On the one hand, anatomical position of surgical scars deeply influences IART location. In patients with a history of atrial septal defect (ASD) and Tetralogy of Fallot repair, the observed macro-rentrant circuits revolving around areas of dense scar or through electrical gaps along double potential lines are generally consistent with the right-sided location of surgical atriotomies [2]. Re-entry around septal patch and left-sided IART have been also observed in rarer cases after ASD correction [2,10]. The electrophysiology substrate is even more complex when Fontan procedure for univentricular hearts is considered [11]. Due to the major hemodynamic abnormalities in these patients, the anatomical location of IART is difficult to predict and depends on the combination of iatrogenic areas of conduction block in heavily remodeled right atrial chambers [11]. However, the classic Fontan (i.e., right atrial to pulmonary artery anastomosis) and the intracardial lateral tunnel were more recently replaced by the so-called extracardiac Fontan where completely external conduits are used. Thanks to a total cavopulmonary connection created through right atrial bypass, the extracardiac Fontan operation has progressively led to a significant reduction in IART occurrence in these patients [12]. In this complex scenario, the implementation of three-dimensional electroanatomic mapping systems proved invaluable in the better understanding of the pathophysiological substrates of these cardiac arrhythmias [11]. Finally, even when typical AFL does represent the predominant arrhythmogenic mechanism, CA is all but straightforward in patients with history of Mustard and Senning correction for congenital transposition of the great arteries. Due to the inherently complex anatomy and presence of an intra-atrial baffle, femoral artery access is often required after atrial switch repair to allow for pulmonary venous atrium mapping retrogradely via the aorta. This approach generally leads to difficult catheter manipulation and stability because of the route tortuosity observed in these cases [2].

### 2.2. Cardiac Surgery for Acquired Heart Disease

**Figure 1:**
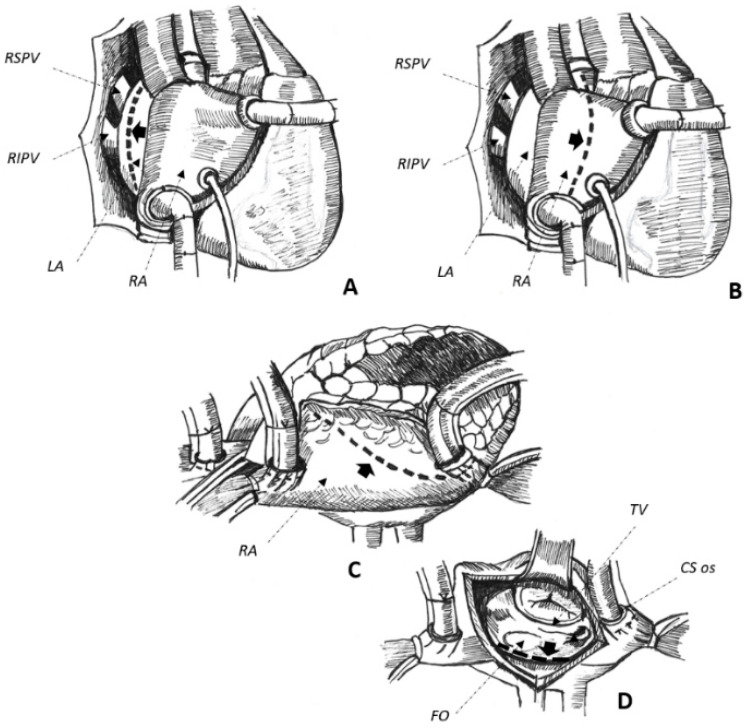
(**A**–**D**). Schematical representation of surgical approaches to get access to the mitral valve. Left atrial approach (**A**), superior trans-septal (**B**), and combined trans-septal approaches, the latter combining a right lateral atriotomy (**C**) with a trans-septal one (**D**) to expose the mitral valve. Atriotomies are reported by interrupted lines marked by thick black arrows in the figure. See text for further details. CS coronary sinus, FO fossa ovalis, LA left atrium, RA right atrium, RIPV right inferior pulmonary vein, RSPV right superior pulmonary vein, TV tricuspid valve.

Cardiac surgery for the correction of mitral valve (MV) disease is common and associated with the development of complex, macro-reentrant arrhythmias revolving around iatrogenic scars [13,14]. In this setting, AAFL is observed in up to 55% of cases [3] and their anatomical location is greatly influenced by atriotomies and cannulation sites performed at the time of surgery [13]. Three major atriotomies have been described for surgical correction of MV disease, as follows: (1) left atrial atriotomy as an incision between the right pulmonary veins and the interatrial septum (Waterston’s groove) (Figure 1A); (2) Guiraudon’s approach or superior trans-septal access involving a vertical right atriotomy extended over the superior right atrium, the septum, and the dome of the left atrium (Figure 1B); and, finally, (3) combined trans-septal approach consistent of a vertical right atriotomy parallel to the atrio-ventricular sulcus (Figure 1C) followed by a separate incision in the interatrial septum (Figure 1D) [15]. On the one hand, trans-septal approaches are more commonly associated with right-sided arrhythmia [13]. Conversely, the anatomical location of AAFL in the left atrium is greatly influenced by left atrial incisions and the concomitant history of surgical ablation of AF. In this setting, the traditional cut-and-sew technique (Cox-Maze-III) and the device-based Cox-Maze-IV procedure are both associated with an increased risk of AAFL [16,17]: electrical conduction gaps through incomplete and non-transmural surgical lesions seem to represent the most common pathophysiological mechanism of the AAFL observed after these surgical procedures [16,17]. Therefore, to set out the proper ablation strategy, it is essential to know which surgical atriotomies were performed at the time of cardiac surgery and whether a Cox-MazeIII/Cox-Maze-IV was carried out in addition to MV valvulopasty/replacement in the investigated patients. In fact, every surgical incision involving the atrial chambers may potentially represent a fertile substrate for the development of re-entrant circuits.

### 2.3. Non-Surgical Pulmonary Vein Isolation

AAFL are common after non-surgical PVI [18]. Independent of the energy source used [19,20], the incidence of AAFL after PVI varies depending on the chosen ablation strategy: from less than 4% after ostial or antral PVI [21,22] to 31% for circumferential pulmonary vein ablation (CPVA) technique [23]. Deployment of ablation lesions in the left atrium is associated with an even greater incidence of AAFL occurrence [4]. Although macro-reentry is the predominant mechanism including left atrial roof and mitral-isthmus-dependent circuits [22], a non-negligible cause of post-PVI AAFL is represented by “small loop” or localized re-entry [4] due to gap-related mechanisms involving reconnected PV [4,22,24]. Although true focal arrhythmias have been rarely described in this setting [25], re-entry as small as 1 cm in diameter could nonetheless be observed after PVI [26]. These circuits usually display multiple slow-conducting channels along their course [27] that show remarkably long fractionated diastolic potentials lasting up to 140 milliseconds [4]. For this reason, at the end of the index CA, pulmonary veins should always be tested for persistent isolation, and ablation lines evaluated to identify conduction gaps. Broadly speaking, testing for electrical isolation requires, at a minimum, validation of an entrance conduction block [28], however, high voltage pacing in the PV may also be considered to evaluate a full bidirectional conduction block through the ablated PV [28]. Furthermore, prolongation of the ablation procedure with various intraprocedural techniques to reveal dormant conduction seems unnecessary in this setting [29]. In fact, as recently assessed in a randomized controlled trial [29], neither adenosine test nor a 30-min waiting phase after PVI showed an improved long-term AF freedom when compared to standard procedure.

### 2.4. Absence of Manifest Structural Heart Disease

In up to 6% of cases, AAFL occurs in patients with no evidence of structural heart disease [5]. Reasons for spontaneous atrial scarring are not clear. However, chronically increased atrial pressure overload in hypertension, occlusion of small coronary artery branches, isolated inflammation, and finally amyloid infiltration may explain an arrhythmogenic substrate in otherwise apparently healthy individuals [5,30]. Most of these circuits are right-sided and usually involve electrical silent areas located at the posterior or the lateral free wall of the right atrium, which can be effectively treated by radiofrequency energy applications delivered from these scars to the inferior vena cava ostium [5,31]. However, narrow and slow-conducting channels may also be found in left-sided, antero-septal circuits, as the result of the complex interweaving of epicardial fibers promoting AAFL [5]. Finally, even in normal hearts, transverse conduction across the crista terminalis [30] and the complex anatomy of the interatrial septum [32] may lead to macro-reentrant arrhythmias due to mechanisms of non-uniform anisotropy [30,33].

## 3. Materials and Methods: Identification of Studies Exploring the Feasibility of Catheter Ablation of Atypical Atrial Flutter in a Wide Spectrum of Heart Diseases

We included studies assessing the major clinical scenarios in which AAFL are commonly observed, such as surgical correction of CHD (IART), cardiac surgery for acquired valvular heart disease, and CA of AF (PVI procedure). Patients with AAFL and no history of structural heart disease were also included. To assess the role of three-dimensional electro-anatomic mapping systems, studies implementing these cutting-edge technologies were compared with those using traditional mapping or a combination of these techniques (i.e., three-dimensional mapping in addition to the traditional one). Therefore, we performed a non-systematic bibliographic research on Medline considering manuscripts published up to and including 2021. For each clinical scenario, the identification and selection of papers was based on the two following criteria: (1) a greater sample size when multiple manuscripts were available and, in case of small sample size papers, (2) a study was included according to its clinical relevance and only after discussion among the authors. The following research MeSH terms helped in the identification of the studies included in Table 1: “Cardiac Arrhythmias”, “Atrial flutter”, “Atrial Fibrillation”, “Catheter Ablation”, and “Cardiac Surgical procedures”. Different combinations of the MeSH terms were used, and the reference list of each paper was analyzed for the identification of further manuscripts of potential clinical interest. The literature research was independently conducted by two authors (RM and JM) and then revised by RM, FA, and JM who reached a shared decision by consensus in case of discordance.

As shown in Table 1, 33 studies [4,5,7,9,10,14,21,31,34,35,36,37,38,39,40,41,42,43,44,45,46,47,48,49,50,51,52,53,54,55,56,57,58] published from 1996 [10] to 2021 [58] were collected. One-thousand-six-hundred-and-forty-one patients undergoing CA for AAFL were analyzed in different clinical settings. For each study, data on the adopted mapping and ablation strategies were collected (conventional mapping relying on intracavitary signals only vs. implementation of three-dimensional mapping systems including high-density mapping tools) together with the identified ablation site (right and/or left atrium), type of ablation catheter used (conventional 4 and 8 mm-tip and/or irrigated-tip catheters with/without contact force sensing technology), peri-procedure feasibility (acute efficacy and development of systemic or groin complications), and other procedure data (procedure and fluoroscopy time where reported). Finally, follow-up data were collected regarding overall follow-up duration, recurrence rate, and overall maintenance of SR on/off antiarrhythmic drugs.

## 4. Overall Peri-Procedure Feasibility: Short-Term Efficacy and Safety

Table 1 displays the evidence from our literature search on the efficacy and safety of CA of AAFL in different surgical, interventional, and clinical settings. The reported studies implemented different mapping strategies, which may have influenced the overall procedural outcome. Although pioneering works based their CA strategy on conventional mapping through the systematic evaluation of transient concealed entrainment and post-pacing intervals at different pacing sites [10,34,35], the feasibility of entrainment is known to be limited due to pacing-mediated arrhythmia termination, degeneration into AF, or increased pacing thresholds in patients on antiarrhythmic medications [31,36,44,46]. Moreover, AAFL are complex arrhythmogenic circuits sustained by double or multiple loops in up to 60% of cases, which would make an ablation strategy based on conventional mapping particularly challenging [10]. To overcome these issues, three-dimensional electroanatomic mapping systems have been progressively implemented in cardiac electrophysiology to guide mapping [36,46,58] and to achieve effective radiofrequency ablation of these complex circuits [7,9,44,46,49,55,57]. In fact, when these systems are used, the peri-procedure success rate spans from 65% [37] to 100% [9,49,53,57], with better results observed in patients with a history of non-surgical PVI or in case of no structural heart disease [48,53,58]. Moreover, by preventing electrode–tissue interface boiling and reducing coagulum and char formation, open-irrigation-tip catheters seem associated with even higher acute success rates with less X-ray exposure and radiofrequency delivery in the setting of AAFL ablation [47].

However, despite the implementation of the latest technologic developments in experienced hands, such as high-density mapping tools [54,56] or contact-force sensing catheters [56], peri-procedure failure is observed in up to 15–20% of cases [54,56], with a greater chance of acute failure in patients with history of surgical correction for CHD [37]. Difficult-to-ablate anatomical substrates [10], peculiar features of the targeted isthmi [51,52], and their anatomical locations [46,59], may explain failures. Further, a CA procedure may also be prematurely interrupted for safety issues to avoid right hemidiaphragm palsy [35], inadvertent block of the atrioventricular node [46], or atrial wall perforation with possible cardiac tamponade. Finally, as shown in Table 1, the inherent complexity of CA of AAFL is proved by the reported long procedure [39] and fluoroscopy times [36].

As for the overall peri-procedure safety, local complications may occur in up to 7% of cases (Table 1), including groin hematoma (up to 7%) [39], arteriovenous fistula (3–4%) [46,51], and femoral pseudoaneurysm (1.4%) [34] in generally anticoagulated patients. On the other hand, regarding systemic complications, cerebral [4,36] and peripheral [35] embolism could be as high as 4–6% with potentially life-threatening major bleedings only sparingly described, including retroperitoneal hemorrhage (2.2%) reported in one study only [3]. Finally, patients with mechanical valve prostheses may portend even a greater risk of peri-procedure thromboembolic or hemorrhagic complications. Therefore, particular attention should be paid to periprocedural antithrombotic regimens in this patient population to avoid potentially life-threatening events [14].

## 5. Maintenance of Sinus Rhythm after a Successful Procedure: Problems Related to the Mid- and Long-Term Clinical Outcome

As displayed in Table 1, AAFL recurrence is observed in up to 62% of cases after a single CA procedure with an overall SR maintenance as low as 38% on/off AAD after a variable follow-up duration, spanning from 7 ± 3 [54] to 37 ± 15 [5] months. Data on whether patients were on AAD before the procedure and at follow-up was not available in most of the studies, and the effect of AAD is therefore unclear in this setting.

The older the publication date, the greater the incidence of arrhythmia recurrence. This would suggest that the recent implementation of dedicated mapping tools [57] and irrigated-tip catheters [42,49] could help the cardiac electrophysiologist to achieve a greater long-term SR maintenance after an initially successful CA procedure [51,57]. The adoption of dedicated, tachycardia-oriented strategies for mapping and ablation of AAFL seem associated with even better results [46,51]. However, the greater the complexity of the atrial substrate to ablate, the higher the incidence of arrhythmia recurrence at follow-up. The worst long-term clinical outcome is commonly seen in patients with surgically corrected CHD (46–52% AAFL recurrence) [34,35], with better results observed after PVI (16–28%) [21,58] or in patients with apparently normal hearts (9–25% of tachycardia recurrence) [5,31].

The beneficial role of multiple CA procedures after AAFL recurrence in achieving long-lasting SR is controversial. In fact, as recently reported by a meta-analysis collecting data on patients with prior surgical correction for MV disease undergoing CA of AAFL, the overall maintenance of SR was significantly lower for repeat procedures compared with history of single CA only (49% vs. 66%, *p* < 0.0001) [14]. Moreover, the longer the follow-up duration, the greater the observed incidence of AF [14]. In fact, the long-term occurrence of this arrhythmia after CA of AAFL could be as high as 21% in different clinical settings [5,36,39,42,49]. These data hint at a progressive atrial substrate modification occurring in these patients that would be associated with the development of complex atrial arrhythmias, AF included, over a mid- and long-term follow-up [14]. To support this hypothesis, investigating the pathophysiological mechanisms in patients with a history of surgically corrected CHD undergoing CA for recurrent AAFL, De Groot et al. [60] observed that most of these circuits would not represent, in fact, true tachycardia recurrence but different arrhythmia morphologies when compared with those mapped and ablated during index CA. Similar observations were also reported in other studies [50,53,55], which would greatly limit the likelihood of long-term maintenance of SR in these patients.

## 6. The Winding Path to Improve the Procedure and the Overall Clinical Outcome: Between Technical and Clinical Aspects

AAFL are typically sustained by critical *isthmi* anatomically [36] and functionally [4,38,39,46] defined. Regardless of the underlying structural heart disease and/or prior iatrogenic scars, these anatomical regions are bounded by anatomical/functional barriers and are associated with low bipolar voltages, fragmented electrograms [39,61], slow conduction velocity [51], and a typical mid-diastolic activation during ongoing tachycardia [10], which make these regions amenable to effective radiofrequency ablation [46].

For these reasons, as already described elsewhere [46,51], the integration of electro-anatomical information provided by these systems with surface and intracavitary signals would allow for the straightforward identification of the mid-diastolic isthmus amenable to radiofrequency ablation for effective arrhythmogenic substrate elimination. However, to avoid any misleading interpretation of the underlying circuit, accurate and high-density mapping of investigated atrial chambers is required to account for almost 90% of the tachycardia cycle length and thereby avoiding missing mapping segments potentially due to non-annotated low-amplitude and fragmented electrograms as low as 0.03–0.05 mV [27]. In this setting, the recent development of new tools, such as Octaray^TM^ system (Biosense Webster Inc., Irvine, CA, USA) and the Ensite^TM^ Omnipolar Technology (OT) (Abbott, Chicago, IL, USA) might allow for even better results [62,63,64], provided that the multitude of signals collected is correctly acquired and interpreted.

Although the implementation of these new technologies in CA procedures seems helpful in most cases, including mapping of re-entrant circuits with multiple loops [45,46], CA of the mid-diastolic isthmus may still be challenging due to its anatomical location and extension [59]. Alternative ablation strategies meant to search for more practical *isthmi* should be considered in specific settings, such as in case of roof-dependent and mitral annular AAFL [46,59].

The ablation of roof-dependent circuits may be particularly challenging. The myocardial musculature surrounding the superior PV is generally thick and consistently displays adipose tissue separating the septopulmonary from the more endocardial septoatrial bundles [65]. This complex interweaving of myocardial fibers and their epicardial course may lead to non-transmural lesions and, thereby, to CA failure [66]. However, an ablation line deployed on the left atrial floor that connects the inferior PV seems a more effective alternative strategy to treat these circuits [66].

As for mitral annular AAFL, a posterior line connecting the left inferior PV to the lateral MV annulus (i.e., posterior mitral isthmus ablation) represents the traditional CA strategy to manage these circuits. However, the remarkable myocardial thickness in this anatomical region; the convective cooling as the result of coronary sinus flow; the epicardial connections; and, not least, the risk of damaging the left circumflex coronary artery may all pose several challenges. To overcome these issues, epicardial ablation from the coronary sinus [67]; endocardial ablation with temporary coronary sinus occlusion to reduce conductive cooling [68]; and, finally, ethanol injection into the Marshall vein [69] are the strategies put forward to achieve the definitive electrical conduction block across the posterior mitral isthmus ablation line. However, alternative ablation lines have been described and may prove helpful in interrupting these circuits, such as the septal mitral isthmus line (connecting the right superior PV to the antero-septal mitral annulus) or the modified anterior line (transecting the myocardial tissue from the anterior aspect of the left atrial appendage to the antero-lateral mitral isthmus) [59,67].

Regardless of the location of the tachycardia circuit or length of the ablative lesion, bidirectional conduction block does represent the essential endpoint of every CA procedure by evidence of detouring of the electrical wavefront around an anatomical barrier or scar through dedicated pacing maneuverers and/or demonstration of double potentials along the performed ablation lines. In some particular cases, demonstration of conduction block with the abovementioned criteria can be difficult and, therefore, complete disappearance of electrical signals at the target site can be considered a surrogate endpoint.

When a macro-reentrant circuit cannot be identified in the atria, a small, localized reentry can be the arrhythmia mechanism, which calls for a more accurate mapping and signal interpretation [4]. Alternatively, a focal activation from a single focus can be identified with a centrifugally spreading wavefront pattern. In fact, although rarely, even in some post-surgical cases, focal atrial tachycardia with a short cycle length resembling AAFL on surface ECG can be observed [70,71]. Interestingly, areas of abnormal conduction can generate not only macro-reentrant circuits, but also fast focal arrhythmias usually observed at the border of a scar area.

Apart from all these technical aspects, clinical considerations are also to be taken into account for proper patient management. First, the clinical history and prior cardiac surgery, particularly the Cox-Maze-IV procedure, should be reviewed in order to obtain information on all potential sites of surgical and non-surgical scars or conduction gaps in the atrial chambers. Second, symptom burden and hemodynamic tolerance of the AAFL should guide the electrophysiologist to assess how aggressive the ablation strategy should be, especially when multiple procedures are required. Third, although surface P wave morphology poorly predicts the arrhythmia origin in this setting, it is important to examine surface ECGs of all the documented episodes to evaluate the presence of multiple morphologies and possibly multiple reentrant pathways. Fourth and even more important, concomitant AF should be diagnosed in advance of CA to evaluate the need for adjunctive pulmonary vein isolation or long-term antiarrhythmic drug therapy. Fifth, pre-procedure imaging with a particular focus on atrial chamber is important to evaluate the substrate and clarify cases with complex anatomy or undiagnosed conditions.

Despite all efforts, AF and other complex atrial arrhythmias refractory to medical therapy may eventually occur even after an initially successful CA procedure as an unavoidable sequela of the progressive electro-anatomic atrial remodeling occurring in diseased atrial chambers [14]. This underlines the importance of proper patient selection and/or early treatment to avoid ablation attempts in already heavily remodeled hearts. In these cases, similarly to AF, atrio-ventricular junction ablation combined with permanent pacemaker implantation (the “ablate and pace” approach) could be an acceptable alternative treatment strategy for symptomatic, drug-refractory AAFL.

## 7. Limitations

The current manuscript is affected by several limitations. First the non-systematic nature of this review. Then, study heterogeneity due to different patient populations undergoing a variety of surgical, non-surgical procedures or, in some cases, even naïve to any prior iatrogenic lesion. Finally, most papers were retrospective and observational in nature. This might have influenced our observations because of potentially uncontrolled confounders and/or other inherent biases affecting this type of study design (e.g., recall bias).

## 8. Conclusions

AAFLs are difficult-to-manage re-entrant arrhythmias that seem amenable to safe and effective CA. In experienced hands, the correct implementation of three-dimensional mapping systems, high-density mapping tools, and a clinically oriented approach could be important to clarify the arrhythmia substrate and plan a rational and effective ablation strategy. Failure to suppress the AAFL or early recurrences are possible, mainly in the presence of an arrhythmogenic substrate unsuitable for CA. Therefore, due to heavily remodeled atria, patients with a history of several surgical or interventional procedures may be prone to develop AAFL with multiple morphologies in addition to other complex atrial arrhythmias, AF included. In fact, in the long-term follow-up, the occurrence of AF could still be expected despite multiple and effective CA procedures. Therefore, a patient-oriented approach is needed for proper patient selection and peri- and post-procedure management. Differently from the setting of surgically corrected congenital heart diseases, patients with AAFL and a history of non-surgical PVI or no prior interventional procedures seem to greatly benefit from CA even at a long-term follow-up.

## Figures and Tables

**Table 1:** Review of current literature on the feasibility of catheter ablation of atypical atrial flutters in different patient populations.

Author	Year	Pts, n.	Age (Years)	Clinical Setting	Prior PVI	Ablation Site	Mapping Strategy	Cath	Acute Success	Systemic Compl	Local Compl	Proc. Time (min)	Fluoro Time (min)	AAFL Recurrence at FU after an Acutely Successful CA Procedure	Overall SR Maintenance at FU after CA	FU (Months)
Kalman, et al. [10]	1996	18	27 ± 15	CHD	0%	RA	Conv.	4 mm	83%	n.r.	n.r.	n.r.	n.r.	27%	61%	17 ± 8
Baker, et al. [34]	1996	14	34 ± 25	CHD	0%	RA	Conv.	4 mm	93%	0%	1.4% (PA)	n.r.	n.r.	46%	50%	8 ± 5
Triedmann, et al. [35]	1997	45	25 ± 11	CHD	0%	RA	Conv.	4 mm	73%	4.4% (PE)	2.2% (GH)	n.r.	n.r.	52%	36%	17 ± 11
Jais, et al. [36]	2000	22	60 ± 14	SHD, MVS	0%	LA	Conv. + 3D	4 mm	77%	4.5% (CE)	0%	339 ± 113	95 ± 42	6%	73%	15 ± 7
Delacretaz, et al. [37]	2001	20	43 ± 15	CHD	0%	RA/LA	Conv. + 3D	4 mm	65%	0%	0%	n.r.	26 ± 9	8%	60%	19 ± 14
Nakagawa, et al. [38]	2001	16	(15–53)	CHD	0%	RA	Conv. + 3D	4 mm	100%	0%	0%	n.r.	n.r.	20%	80%	13
Ouyang, et al. [39]	2002	28	64 ± 10	VHS, SHD	n.r.	LA	Conv. + 3D	4 mm, IC	88%	0%	7% (GH)	384 ± 145	18 ± 9	0%	71%	14
Zrenner, et al. [40]	2003	12	29 ± 5	CHD	0%	RA	Conv. + 3D	8 mm	83%	0%	0%	343 ± 141	64 ± 43	30%	58%	19 ± 8
Tai, et al. [41]	2004	15	61 ± 13	RHD	0%	RA	Conv. + 3D	4 mm	87%	0%	0%	n.r.	n.r.	15%	67%	17 ± 4
Tanner, et al. [42]	2004	36	(9–67)	CHD	0%	RA/LA	Conv. + 3D	IC	87%	0%	2.7% (AVF)	115 (45–315)	12 (4–56)	8%	78%	17 ± 7
Lukac, et al. [43]	2005	83	(9–73)	CHD, SHD	0%	RA/LA	Conv. + 3D	4,8 mm, IC	88%	1.2% (CE)	0%	135 (27–382)	17 (2–83)	24%	60%	27 (2–76)
Stevenson, et al. [31]	2005	8	53 ± 12	No SHD	0%	RA	Conv. + 3D	4 mm, IC	87%	n.r.	n.r.	n.r.	n.r.	25%	75%	20 ± 13
Magnin-Poull, et al. [44]	2005	22	43 ± 12	CHD	0%	RA	Conv. + 3D	4 mm	100%	0%	0%	290 ± 155	24 ± 12	54%	41%	25 ± 16
Deisenhofer, et al. [4]	2006	16	58 ± 8	PVI	100%	LA	Conv. + 3D	IC	89%	6% (CE)	0%	283 ± 66	47 ± 22	62%	38%	10 ± 7
Seiler, et al. [45]	2007	40	52 ± 12	CHD, VHS	0%	RA/LA	Conv. + 3D	IC	88%	n.r.	n.r.	n.r	n.r	37%	55%	28 ± 17
De Ponti, et al. [46]	2007	65	57 ± 17	CHD, SHD	9%	RA/LA	3D only	4,8 mm, IC	92%	0%	3% (AVF)	n.r	n.r	6.8%	80%	14 ± 4
Fiala, et al. [5]	2007	33	62 ± 11	No SHD	0%	RA/LA	Conv. + 3D	4 mm, IC	84%	0%	0%	191 ± 50	22 ± 9	9%	73%	37 ± 15
Bai, et al. [47]	2007	70	45–71	SHD, PVI	61%	RA/LA	Conv. + 3D	8 mm, IC	86%	0%	0%	150–366	44–116	17%	75%	10
Chae, et al. [48]	2007	78	62 ± 11	PVI	100%	LA	Conv. + 3D	8 mm, IC	85%	0%	0%	n.r.	n.r.	23%	77%	13 ± 10
Esato, et al. [49]	2009	26	59 ± 12	SHD, CHD	73%	RA/LA	Conv. + 3D	IC	100%	0%	0%	181 ± 58	37 ± 19	8%	88%	11 ± 3
Yap, et al. [50]	2010	130	40 ± 13	CHD	0%	RA	Conv. + 3D	4,8 mm, IC	63%	3.3%	n.r.	185–240	42–47	48%	43%	44
De Ponti, et al. [51]	2010	52	54 ± 16	SHD, PVI	17%	RA/LA	3D only	IC	90%	0%	4% (AVF)	n.r	n.r	6%	92%	26 ± 18
Drago, et al. [52]	2011	31	26 ± 17	CHD	0%	RA	3D only	4,8 mm, IC	87%	0%	0%	293 ± 104	38 ± 23	0%	n.r.	12 ± 4
Wasmer, et al. [21]	2012	25	59 ± 10	PVI	100%	LA	Conv. + 3D	IC	n.r.	n.r	n.r	n.r	n.r	28%	64%	31 ± 17
Zhang, et al. [53]	2013	10	57 ± 14	No SHD	0%	LA	Conv. + 3D	IC	100%	0%	0%	n.r	n.r	20%	80%	14 ± 10
Scaglione, et al. [9]	2014	46	49 ± 13	CHD	0%	RA/LA	Conv. + 3D	4,8 mm, IC	100%	0%	0%	110 ± 30	30 ± 9	24%	76%	7 ± 4
Anter, et al. [54]	2016	20	62 ± 7	CA	95%	RA/LA	HD 3D map (Orion^TM^, Boston Scientific, Natick, MA, USA)	IC	80%	0%	5% (GH)	n.r	n.r	25%	75%	7 ± 3
Grubb, et al. [55]	2019	140	45 ± 1	CHD	0%	RA/LA	Conv. + 3D	8 mm, IC	89%	1% (AVB; CE)	n.r.	n.r.	30 ± 2	50%	56%	49.9
Marazzato, et al. [14]	2020	227	49–72	MVS, CM IV	56%	RA/LA	Conv. + 3D	n.r.	96% (87–100)	<1% (RPH; CE)	n.r.	70–306	9–64	n.r.	59% (47–71)	1–63
Derval, et al. [7]	2020	132	60 ± 12	PVI, no SHD	84%	RA/LA	HD 3D map (Orion^TM^, Boston Scientific)	IC	92%	n.r.	n.r.	n.r.	n.r.	46%	54%	13 ± 9
Balt, et al. [56]	2021	23	66 ± 5	HS, PVI	n.r.	RA/LA	HD 3D map (Advisor ^TM^ HD grid, Abbott, Chicago, IL, USA)	IC + CF	84%	4% (CE)	0%	145 ± 42	25 ± 12	21%	75%	12
Liu, et al. [57]	2021	31	59 ± 10	HS, CA	60%	RA/LA	Conv. + HD (Pentaray^TM^, Biosense Webster Inc., Irvine, CA, USA)	IC	100%	n.r.	n.r.	n.r.	n.r.	7%	93%	6
Vlachos, et al. [58]	2021	107	66 ± 9	PVI	100%	LA	Conv + HD (Orion^TM^, Boston Scientific; Advisor^TM^ HD grid, Abbott; Pentaray^TM^, Biosense Webster Inc.)	IC	99%	0%	1% (AVF)	214 ± 90	28 ± 20	16%	84%	16 ± 3

Abbreviations. 3D map = mapping based on 3D-electroanatomic mapping systems; AVB = atrioventricular block; AVF = femoral arteriovenous fistula; Cath = catheter used; CA = prior catheter ablation procedures; CE = cerebral embolism; CF = contact-force sensing catheters; CHD = surgical correction for congenital heart disease; Conv = conventional mapping; CM IV = prior Cox-Maze-IV procedure; Compl = complications; Fluoro = fluoroscopy; FU = follow-up; GH = groin haematoma; HD = high-density mapping tools; HS = prior heart surgery; IC = irrigated catheters; LA = left atrium; MVS = history of mitral valve surgery; n.r. = not reported; PA = femoral pseudoaneurysm; PE = peripheral embolisms; Proc = procedure; Pts = patients; PVI = pulmonary vein isolation; RA = right atrium; RHD = rheumatic heart disease; RPH = retroperitoneal haemorrhage; SHD = presence of structural heart disease; SR = sinus rhythm; VHS = prior valvular heart surgery.
